# Is Off-label repeat prescription of ketamine as a rapid antidepressant safe? Controversies, ethical concerns, and legal implications

**DOI:** 10.1186/s12910-016-0087-3

**Published:** 2016-01-14

**Authors:** Melvyn W. Zhang, Keith M. Harris, Roger C. Ho

**Affiliations:** National Addiction Management Service, Institute of Mental Health, 10 Buangkok Green Medical Park, 539 747 Singapore, Singapore; School of Psychology, University of Queensland, Queensland, Australia; Department of Psychological Medicine, Yong Loo Lin School of Medicine, National University of Singapore, Singapore, Singapore

**Keywords:** Addiction, Autonomy, Consequentialism, Ethical relativism, Fidelity, Ketamine, Nonmaleficience, Off-label, Rapid antidepressant

## Abstract

**Background:**

Depressive disorders are a common form of psychiatric illness and cause significant disability. Regulation authorities, the medical profession and the public require high safety standards for antidepressants to protect vulnerable psychiatric patients. Ketamine is a dissociative anaesthetic and a derivative of a hallucinogen (phencyclidine). Its abuse is a major worldwide public health problem. Ketamine is a scheduled drug and its usage is restricted due to its abuse liability. Recent clinical trials have reported that ketamine use led to rapid antidepressant effects in patients suffering from treatment-resistant depression. However, various flaws in study designs, and possible biased reporting of results, may have influenced those findings. Further analyses of ketamine use are needed to ensure patient safety.

**Discussion:**

The use of ketamine in research and treatment of depressive disorders is controversial. Recently, mental health professionals raised ethical concerns about an ongoing ketamine trial in the UK. Also, a Canadian agency reviewed the existing evidence and did not recommend prescribing ketamine to treat depressive disorders. Findings obtained from tightly controlled research settings cannot be easily translated to clinical practice as substance abuse is commonly comorbid with depressive disorders. An effective antidepressant should reduce severity of depressive symptoms without liability problems. Although the US FDA has not approved the use of ketamine to treat depressive disorders, some psychiatrists offer off-label repeat prescription of ketamine. Prescribing ketamine for treating depressive disorders requires substantial empirical evidence. Clinicians should also consider research findings on ketamine abuse. Depressive disorders can be chronic conditions and the current evidence does not rule out the risk of substance abuse after repeat prescription of ketamine. Off-label ketamine use in treating depressive disorders may breach ethical and moral standards, especially in countries seriously affected by ketamine abuse. This article presents two real-world clinical vignettes which highlight ethical principles and theories, including autonomy, nonmaleficience, fidelity and consequentialism, as related to off-label ketamine use.

**Conclusion:**

We urge clinicians to minimise the risk of harming patients by considering the empirical evidence on ketamine properties and attempting all standard antidepressant therapies before considering the off-label use of ketamine.

## Introduction

### Ketamine as an anaesthetic

Ketamine is a rapid-acting non-barbiturate dissociative anaesthetic. It produces an anaesthetic state characterized by profound analgesia, with normal pharyngeal-laryngeal reflexes. Ketamine was approved by the US FDA as an anaesthetic for cardiac catheterization, skin grafting, orthopaedic and extraperitoneal procedures, as well as diagnostic procedures performed on the eye, ear, nose and throat [1]. Ketamine has been used as a battlefield anaesthetic because it can induce a dissociative state, which is helpful for treating wounded soldiers by maintaining consciousness while they are cognitively separated from pain [2]. Ketamine does, however, have side effects. Common side effects observed during one-time anaesthetic use include: elevation of blood pressure and heart rate, hallucinations, delirium, and irrational behaviours [1]. Common side effects after ketamine infusion included anxiety, confusion, dizziness, drowsiness, intense euphoria, perceptual disturbances, hypertension, nausea and an increase in skeletal muscle tone. Moreover, the current evidence on the safety of ketamine as an anaesthetic applies only to medical or surgical patients for one-time usage, not to psychiatric patients who receive multiple infusions. These adverse effects are usually transient in nature, lasting from a few hours to 24 h. The FDA recommends administering low-dose ketamine via the intramuscular route to reduce the occurrence of adverse effects [1]. Of importance, the FDA also recommends the minimization of verbal, tactile and visual stimulation when ketamine is administered.

### Ketamine abuse

In some countries, ketamine is the most common drug of abuse and the prevalence of health and social problems associated with ketamine abuse are widely acknowledged [3, 4]. In the 1970s, some individuals started misusing ketamine. Ketamine is pharmacologically similar to phencyclidine (PCP), a commonly abused hallucinogen. The street name of ketamine is “K” or “Special K.” Ketamine abuse is now common worldwide. For example, in 2012, 1.5 % of US 12^th^ graders reported ketamine abuse [5]. Emergency room visits due to ketamine abuse increased by 2000 % between 1995 and 2002 in the US [6]. In the UK, the prevalence of ketamine abuse among recreational drug users increased from 25 to 40 % from 2002 to 2007 [7]. In Hong Kong, ketamine is the most common drug of abuse and is consumed by more than 80 % of drug users [3].

Drug abusers may snort ketamine powder or inject liquid ketamine to experience perceptual changes, dissociation and hallucinatory effects. At low doses, ketamine can cause euphoria, sensory distortions, impairments in set-shifting, and heightened feelings of empathy [8–10]. At high doses, ketamine can cause dissociative effects, hallucinations, intoxication, and frightening experiences [2]. Frequent ketamine use has been associated with amnesia, dependence, dissociation, lower urinary tract dysfunction, and poor impulse control [3]. The current legal status of ketamine varies from country to country. In the US, ketamine is a Schedule III drug under the Controlled Substances Act, which limits control in prescription due to its abuse liability [5]. US physicians cannot authorise more than five refills of ketamine per prescription. When ketamine tablets are distributed by prescription, the medication bottle must state that it is a crime to distribute ketamine to others. Individuals convicted of illicit possession of ketamine are subject to imprisonment and/or large fines. Other countries have also rescheduled ketamine to exert tighter control due to its abuse liability and other harmful consequences. Ketamine is a Class B drug in the UK and a schedule I drug in Hong Kong. In Malaysia and Singapore, anyone caught with ketamine faces imprisonment or strokes of the cane [11]. Repeated offenders may face the death penalty in Malaysia.

### Major depressive disorder and existing treatment

Major depressive disorder is highly prevalent and causes significant disability worldwide [12]. Current evidence indicates that 70–90 % of depressed patients respond to initial antidepressant treatment [13]. For 70 % of the patients with treatment resistant depression, combining antidepressants, augmentation with mood stabilisers, integrating antidepressant drugs with psychosocial therapies (such as cognitive behavioural therapy), and offering electroconvulsive therapy (ECT), have been show to lead to recovery [14].

## Discussion

### Clinical trials evaluating ketamine as an antidepressant

Several clinical trials, using slow intravenous (IV) infusion of ketamine at sub-anaesthetic doses, explored its effect as a rapid antidepressant. Response rates have ranged from 29 % [15] to 79 % [16]. Rapid antidepressant states from ketamine were found to be unsustainable, with relapse rates as high as 73 % at one month post-treatment [17]. In addition, most of these studies had inadequate statistical power due to small sample sizes. Therefore, Wan et al. [18] combined results of 97 patients who received short-term IV ketamine (an average of 2.1 infusions per patient, with a mean follow-up period of 2.9 years after the last infusion), and concluded that ketamine treatments were safe and well tolerated. Most published clinical trials of ketamine as an antidepressant included only depressed patients without a history of substance abuse, which is uncommon in the clinical population. Therefore, those findings may not be generalisable as there is insufficient data to demonstrate safe prolonged use of ketamine [18]. Several researchers and their affiliated medical schools declared that they own patents for using ketamine to treat depression and would therefore benefit financially if the FDA approves its use in treating depressive disorders [19–21]. Therefore, some researchers may have been motivated by financial interests.

### Off-label repeat prescription of ketamine

There are clinicians offering off-label usage of ketamine as a treatment option for treatment resistant depression [22]. Off-label usage refers to the prescription of a medication to treat a condition which has not been approved by the FDA, and there is insufficient medical evidence to support such application [23]. Due to the low remission rate and short half-life of ketamine, researchers have advocated multiple ketamine infusions to maintain its antidepressant effect [15, 24]. In the US, some clinics are charging hundreds of dollars for off-label ketamine infusions that have to be administered repeatedly [22]. Rasmussen [25] stressed that the enthusiasm of advocating repeated infusions of ketamine to maintain its antidepressant effect must be tempered by its addiction liability and tolerance effects. Off-label repeat prescription of ketamine may cause potential harm to depressed patients as depression and addiction are commonly comorbid [26]. More concern should be given to the fact that addictive substances can be poor choices for treating vulnerable populations.

### Criticisms of an ongoing ketamine trial

Recently, a controversial longitudinal trial, funded by the National Institute for Health Research (UK), which combines ECT and ketamine treatment, was approved, with recruitment currently underway [27]. The researchers hypothesise that ketamine could reduce the cognitive impairments associated with ECT [28]. However, frequent use of ketamine also leads to cognitive impairments in working memory and executive function, and there is no previous evidence that ketamine enhances cognitive function [29]. The administration of ketamine with ECT is not novel. In 1972, Coppel and Dundee [30] expressed concern that combining ketamine and ECT led to long recovery periods, unpleasant dreams and delirium, which did not occur with conventional anaesthesia. More recently, nine British mental health professionals wrote an open letter to the investigators of the ECT-ketamine trial to express their ethical concerns [31]. For example, they believed the participant information sheet contained misleading information. The study investigators claimed that “ketamine is believed to work together with ECT to improve mood” which the mental health professionals found may be misleading to patients suffering from chronic depression. The open letter urged the investigators to add a qualifier stating that such antidepressant effects are “short-term” or “temporary.” We believe that it is important to inform research participants that two-thirds of patients relapsed within 1 week after IV ketamine infusion and not all patients suffering from major depressive disorder would respond to IV ketamine [32]. Their requests were, however, ignored as was a proposal to suspend the clinical trial.

### Critical appraisal of current evidence

Despite preliminary data supporting the off-label usage of ketamine as a rapid antidepressant, concerns pertaining to these clinical trials have been raised. In 2014, the Canadian agency for Drugs and Technologies in Health reviewed previous ketamine studies and concluded there is a lack of evidence to recommend ketamine to treat depressive disorders [33]. That report identified flaws in previous trials. For example, there remains a lack of direct comparisons between ketamine and other antidepressants, therefore it remains unknown whether ketamine is superior, equivocal or inferior to validated medications. Recently, several clinical trials compared the effects of ketamine and midazolam on depression [20] and suicidal ideation [34]. Midazolam is a benzodiazepine and commonly used for sedation prior to surgical procedures. Midazolam is not an antidepressant and not recommended by any treatment guideline to treat depressive disorders. It is not surprising that ketamine demonstrated greater efficacy due to its acute mood elevation effect. Another limitation is that most studies did not specify previous treatment options, including psychosocial interventions, and depressed participants might have been undertreated, resulting in false resistance to conventional treatment. Also, in one study, the doses of other psychiatric medications (e.g. antidepressant, mood stabilisers) were increased among ketamine responders during the study period [15]. It remained unclear whether treatment responses were due to ketamine or to an increase in doses of other psychiatric medications.

**Fig. 1:**
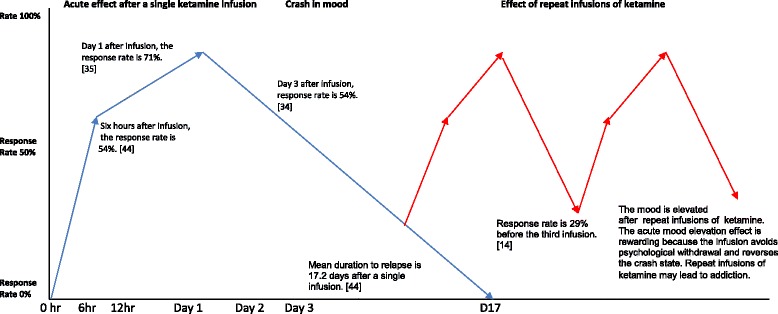
Effects after a single ketamine infusion (*blue line*) and effects of repeat infusions of ketamine (*red line*)

Another research limitation is that participants were monitored for a short period (from 230 min [35] to 21 days [36]) and these studies failed to provide reliable estimates of the risks of ketamine if given repeatedly over a longer period of time. Despite very limited empirical evidence for using ketamine outside of research settings [37], some researchers have advocated repeated ketamine infusions to maintain its antidepressant effect due to its short half-life [15, 24]. Some researchers have emphasised the safety of ketamine at sub-anaesthetic doses [18], and distinguish its effects from the high doses used by recreational users. Others claim that ketamine does not cause dependence with multiple infusions [15], although that is not supported by current evidence. Sub-anaesthetic doses of ketamine are associated with psychomotor activation, which increases the addictive liability of ketamine [2], according to the psychomotor stimulant theory of drug reward [38]. Furthermore, the rapid antidepressant effect of ketamine is not unique and most psychedelic drugs, such as amphetamines, demonstrate acute mood elevation effects. In the 1930s, amphetamines were also believed to be a powerful antidepressant [39]. Their acute mood elevation effect is, however, short-lived and amphetamine takers fall into depressive states shortly after stopping use [40] (see Fig. 1). That same phenomenon is commonly observed after a single ketamine infusion. Patients may try to avoid mood crashes or withdrawal by requesting more ketamine infusions. The acute mood elevation effect is rewarding and causes classical substance addiction. The potential addiction associated with long-term ketamine use is described as the “slippery ketamine slope” [41], because patients will slip into addiction and the field of psychiatry will slip into the unethical practice of using inappropriate drugs to treat psychiatric conditions, resulting in serious ethical concerns [4]. Sanacora and Schatzberg [42] also objected to using ketamine to treat depressive disorders and described this as a potential “false prophecy”. Many experts argue that off-label intermittent ketamine infusions may cause harm and addiction, which does not adhere to the principle of non-maleficence. In some countries, ketamine is the most common drug of abuse and the prevalence of health and social problems associated with ketamine abuse is widely acknowledged [3, 43]. The off-label repeat prescription of ketamine may be less morally acceptable in countries where ketamine abuse is a serious public health problem. The theory of ethical relativism explains variances in prevalence of ketamine abuse in different countries which will influence the perception and acceptance of the off-label use of ketamine.

### Case vignettes illustrating ethical concerns related to off-label repeat prescription of ketamine

We present two hypothetical case vignettes, based on our clinical experiences and knowledge, to highlight possible ethical concerns surrounding off-label repeat prescription of ketamine.

### Case vignette 1

A 60 year-old man suffering from severe treatment resistant depression presented to an outpatient psychiatrist office requesting ECT. Previously, he did not respond to treatment with selective serotonin reuptake inhibitors (SSRI), serotonin noradrenaline reuptake inhibitors (SNRI), tricyclic antidepressants (TCA), or monoamine oxidase inhibitors (MAOI). The attending psychiatrist decided, therefore, to try off-label repeat infusions of ketamine, informing the patient that conventional ECT administered to bilateral temporal regions would cause severe memory impairment and has less efficacy compared with ketamine. The psychiatrist claimed that ketamine is very safe, with fewer side effects than ECT. The psychiatrist asked the patient to stop other antidepressants, without explaining the unknown risks and the unknown course of ketamine. The patient’s depression improved shortly after ketamine infusions but relapsed two weeks after treatment. The psychiatrist suggested continuing ketamine infusions on a fortnightly basis and charged him US$200 per treatment. After three years of treatment, the patient developed urinary incontinence, which was determined to possibly be caused by frequent ketamine infusions.

### Discussion of case vignette 1

#### Autonomy

Consideration should be given to patient treatment choices and preferences. In this case vignette, the psychiatrist declined the patient’s request for ECT. This scenario is common and we have encountered this situation in our clinical practice. Similarly, in New York, there were reports of patients suffering from severe depressive disorders who requested ECT but were persuaded to receive ketamine infusions instead [14]. Those patients showed minimal improvement after infusions of ketamine and were later administered ECT. The off-label use of ketamine only delayed their eventual treatment. In this case vignette, the psychiatrist failed to offer alternative treatments, such as unilateral ECT, which causes less cognitive impairment [44]. ECT has been extensively evaluated and recommended by the American Psychiatric Association (APA) as a conventional treatment in depressive disorders [44]. In contrast, ketamine is an experimental treatment under investigation and evaluation. The National Commission for the Protection of Human Subjects of Biomedical and Behavioral Research established a clear boundary between trial and conventional treatment, and emphasised the need for informed consent. The psychiatrist did not demonstrate respect for the patient’s personal rights when he blurred the boundaries between conventional psychiatric treatment and off-label repeat prescription of ketamine.

### Fidelity and financial conflict of interest

In the above vignette, the patient was denied conventional treatments (i.e. ECT and SSRI) and was persuaded to consider off-label ketamine treatment. Furthermore, there was a potential financial conflict of interest, as the psychiatrist charged thousands of dollars for repeat off-label infusions of ketamine, much more than the costs of alternative treatments. The psychiatrist did not exercise fidelity which is based upon the virtue of caring. It is also possible that the doctor prescribed repeated ketamine for personal financial gain.

### Informed consent and nonmaleficience

In the vignette, the psychiatrist did not inform the patient of the empirical evidence of the possibility of side effects, such as urinary incontinence associated with long term use of ketamine. Instead, he presented one-sided information about ketamine and ECT. Previous studies reported dissociative and psychotomimetic effects after ketamine infusions, explained above [35]. Such biased information could have a detrimental effect on depressed patients. There is also evidence that depression lowers a person’s capabilities and perceived self-efficacy [45]. As a result, depressed patients can be vulnerable to suggestion, especially when they are desperate for cure. In this case vignette, the psychiatrist did not appear to adhere to the principles of informed consent and non-maleficence.

### Legal implications

As the vignette psychiatrist did not fulfill his fiduciary duty, the patient could take legal action for the resulting urinary incontinence. It may lead to class action suits if more patients suffer from urinary incontinence due to frequent ketamine infusions. Fiduciary duty is a legal obligation of doctors to act in the best interests of their patients. The patient placed total trust and confidence in the psychiatrist to manage his depressive disorder and protect him from harm. This case could be considered medical negligence as it fulfils all three components of Bolam’s test. The psychiatrist had a duty of professional care to the patient, but as a consequence of a breach of that duty, the patient suffered harm.

### Case vignette 2

A 30-year-old woman with treatment resistant depression and a history of ketamine abuse presented to a psychiatric clinic for the first time after discharge from a hospital for suicidal ideation. She reported that she had been on several different antidepressants that had failed, including SSRI, TCA, and MAOI, but she did not report her past history of ketamine and hallucinogen abuse. The patient requested ketamine as she heard from the media that ketamine is a safe and rapid antidepressant which could reduce suicidal thoughts. The psychiatrist agreed to prescribe ketamine to treat her depression and suicidal ideation. He did not obtain collateral information from her previous health providers. Subsequently, the patient came to the clinic frequently and asked for ketamine refills. The psychiatrist agreed to issue six refills of ketamine in one prescription. Six months later, the patient was arrested by police as her urine sample was positive for ketamine and she was involved in international smuggling of ketamine. She could not recall whether the ketamine was prescribed by her psychiatrist or purchased abroad. She thought that ketamine was a safe antidepressant and had been taking ketamine on a daily basis to avoid mood crashes. She also distributed ketamine to friends. She is facing criminal charges of conspiracy to distribute ketamine, which carries a penalty of 5 years in prison.

### Discussion of case vignette 2

#### Capacity to give consent

For case 2, the debate is whether the patient had the capacity to consent to off-label ketamine treatment as she had a history of substance abuse, including ketamine abuse. Some ethicists argue that individuals can have reduced ability to resist desires for a drug of choice, limiting their capacity to give consent [4]. Abusers of ketamine and other substances are therefore likely to differ from patients without a history of substance abuse. The harm they may suffer could be considerable if their consent is accepted as valid without assessing past history of substance abuse.

### Non-maleficence

The psychiatrist of this vignette did not obtain collateral information from previous health providers. The patient continued her addiction to ketamine with the assistance of the psychiatrist. She developed withdrawal symptoms (i.e. mood crashes) when she ran out of ketamine. As a result, she asked for frequent ketamine refills which caused additional harm (i.e. addiction and legal consequences). The psychiatrist was negligent in not inquiring on the patient’s history of substance abuse and, therefore, did not adhere to the principle of non-maleficence.

Paying special attention to past substance abuse and forensic history is an integral part of psychiatric assessment. Most of the clinical trials studying the efficacy of ketamine in treating major depressive disorder excluded participants with histories of substance abuse, and claimed that ketamine did not cause dependence with multiple infusions after a short period of observations [15]. Can the possibility of addiction be completely ruled out if ketamine is prescribed repeatedly over a prolonged period? We believe that it cannot. To see how addiction could develop, consider the properties of ketamine. Ketamine has high first past metabolism [46] and a short half-life (3 h) [47]. Ketamine does not maintain acute mood elevations, therefore, patients can crash into depressive states shortly after stopping use [39]. Users try to avoid crashes by taking more of the substance. The acute mood elevation effect is rewarding and can lead to drug addiction. As a result, off-label repeat prescription of ketamine is not likely to be safe and may cause substantial harm.

### The consequentialist ethical theory

An additional argument is based on the consequentialist ethical theory, which considers an act as ethical or unethical depending on the consequences of the act. Based on consequentialism, the off-label repeat prescription of ketamine would be unethical for the above patient because it continued her addiction and led to her arrest. In this case, the patient was misled by media reports which described ketamine as a safe and rapid antidepressant. The British Medical Journal published an article in 2015 which stated the media should not just report artificial mood elevation effects of ketamine but also report the risk of abuse, which is a concern for psychiatric patients [48]. Some academic institutions, however, have issued inflated claims of their own research results on open-label ketamine trials in order to attract media coverage [49]. Press releases on ketamine frequently contain sensational and unsubstantiated titles such as “Ketamine: The future of depression treatment” [50] or “Rave drug holds promise for treating depression” [51] without highlighting its potential for abuse, and the liability and complications of long-term use. Media biases may not be apparent to many patients, making it imperative that doctors fully inform them of all treatment effects and possibilities. Ketamine abusers can have a false perception that ketamine is relatively safe. Media reporting guidelines may help improve patient safety by better informing them of the potential side effects, long-term effects, and other consequences of using drugs like ketamine.

### Legal implications

From a legal perspective, the psychiatrist in the vignette violated the Controlled Substances Act by issuing more than five refills of ketamine without consideration of the patient’s history of substance abuse and her interactions with other illicit drugs [5]. The psychiatrist failed to consider potential legal implications of prescribing ketamine to patients who might have been involved in trafficking of ketamine from illegal sources.

## Conclusions

Ethical principles and medical research are integral to the delivery of safe and effective psychiatric treatment. The off-label use of ketamine as an antidepressant requires open debate and further research. We conclude that ketamine may not be a safe antidepressant due to the following reasons:

- No other antidepressant behaves as an antidepressant at low doses but a drug of abuse at high doses. In other words, there is no scientific evidence showing a drug has two different properties based on dose levels (i.e. a rapid antidepressant at low doses and a drug of abuse at high doses).
- The current data on ketamine safety is based on small numbers of infusions and short periods of observations. Some researchers claim that ketamine does not cause addiction when it is used at a sub-anaesthetic dose to treat depressive disorders. Allowing such claims to remain unchallenged or to be accepted as sufficient evidence, could be considered unethical treatment of patients.

As the possibility of ketamine abuse cannot be ruled out by clinical trials conducted in tightly controlled research environments, clinicians need to consider the empirical evidence on the abuse liability of ketamine and its complications before considering off-label repeat prescription. In our view, patients may have inadequate understanding of the potential harm (e.g. urinary incontinence, addiction) associated with long-term ketamine use, especially as a maintenance treatment for depressive disorders. Clinicians must inform their patients of both the positive and negative aspects of ketamine. Assessment of history of substance abuse should also be compulsory before any off-label prescription of ketamine is made. Patients with histories of substance abuse should probably not receive ketamine to minimise risks to those in care. We believe clinicians need to be more aware of the legal status of ketamine and the impact of ethical relativism which could affect the acceptance of ketamine in society, especially in countries where ketamine abuse poses a serious threat to public health. We therefore suggest that clinicians should minimise the risk of inflicting harm to patients by considering the ethical principles, the properties of ketamine, and attempting all standard antidepressant therapies before considering off-label repeat prescription of ketamine.

## Declarations

The authors (MWZ and RCH) have encountered questionable practices in research and clinical service related to using ketamine to treat depressive disorders. The authors hope to inform other academics and clinicians of the importance of developing a full understanding of ketamine to minimise harm to patients. The authors urge academic institutions and hospitals to uphold ethical and safety concerns related to ketamine research and its off-label use.
